# *SLC12A* ion transporter mutations in sporadic and familial human congenital hydrocephalus*

**DOI:** 10.1002/mgg3.892

**Published:** 2019-08-08

**Authors:** Sheng Chih Jin, Charuta G. Furey, Xue Zeng, August Allocco, Carol Nelson‐Williams, Weilai Dong, Jason K. Karimy, Kevin Wang, Shaojie Ma, Eric Delpire, Kristopher T. Kahle

**Affiliations:** ^1^ Department of Genetics Yale University School of Medicine New Haven CT USA; ^2^ Laboratory of Human Genetics and Genomics The Rockefeller University New York NY USA; ^3^ Department of Neurosurgery Yale University School of Medicine New Haven CT USA; ^4^ Department of Anesthesiology Vanderbilt University School of Medicine Nashville TN USA; ^5^ Department of Cellular & Molecular Physiology Yale University School of Medicine New Haven CT USA; ^6^ NIH‐Yale Centers for Mendelian Genomics, Yale School of Medicine New Haven CT USA

**Keywords:** hydrocephalus, KCC3, KCC4, *SLC12A6*, *SLC12A7*, whole exome sequencing

## Abstract

**Background:**

Congenital hydrocephalus (CH) is a highly morbid disease that features enlarged brain ventricles and impaired cerebrospinal fluid homeostasis. Although early linkage or targeted sequencing studies in large multigenerational families have localized several genes for CH, the etiology of most CH cases remains unclear. Recent advances in whole exome sequencing (WES) have identified five new *bona fide* CH genes, implicating impaired regulation of neural stem cell fate in CH pathogenesis. Nonetheless, in the majority of CH cases, the pathological etiology remains unknown, suggesting more genes await discovery.

**Methods:**

WES of family members of a sporadic and familial form of severe *L1CAM* mutation‐negative CH associated with aqueductal stenosis was performed. Rare genetic variants were analyzed, prioritized, and validated. De novo copy number variants (CNVs) were identified using the XHMM algorithm and validated using qPCR. *Xenopus* oocyte experiments were performed to access mutation impact on protein function and expression.

**Results:**

A novel inherited protein‐damaging mutation (p.Pro605Leu) in *SLC12A6*, encoding the K^+^‐Cl^−^ cotransporter KCC3, was identified in both affected members of multiplex kindred CHYD110. p.Pro605 is conserved in KCC3 orthologs and among all human KCC paralogs. The p.Pro605Leu mutation maps to the ion‐transporting domain, and significantly reduces KCC3‐dependent K^+^ transport. A novel de novo CNV (deletion) was identified in *SLC12A7*, encoding the KCC3 paralog and binding partner KCC4, in another family (CHYD130) with sporadic CH.

**Conclusion:**

These findings identify two novel, related genes associated with CH, and implicate genetically encoded impairments in ion transport for the first time in CH pathogenesis.

## 1 INTRODUCTION

Congenital hydrocephalus (CH), the primary enlargement of the cerebrospinal fluid (CSF)‐filled brain ventricles, affects 1/1,000 births and is treated with empiric surgical CSF diversion techniques with substantial morbidity (Kahle, Kulkarni, Kulkarni, Limbrick, & Warf, 2016; Munch et al., 2012; Simon et al., 2008). However, significant gaps in our understanding of the molecular pathogenesis of CH impede the development of preventive, diagnostic, and therapeutic measures. Although ~40% of all CH cases are predicted to have a genetic etiology, our knowledge of specific CH‐causing genes and their pathogenic mechanism remains primitive. Notably, although early linkage or targeted sequencing studies in large multiplex CH families have found four risk genes, including *L1CAM* (OMIM# 307000) (Rosenthal, Jouet, Jouet, & Kenwrick, 1992), *CCDC88C* (OMIM# 236600) (Ekici et al., 2010), *MPDZ* (OMIM# 615219) (Al‐Dosari et al., 2013), and *AP1S2* (OMIM# 300629) (Tarpey et al., 2006), these findings in aggregate explain less than 5% of CH cases, suggesting additional risk genes await discovery.

Fundamental obstacles to accelerating CH gene discovery include locus heterogeneity, phenotypic complexity, and the sporadic nature of most CH cases, limiting the utility of traditional genetic approaches in the past. Recent advances in next‐generation sequencing technologies have revolutionized approaches for understanding the genetics of complex human diseases, enabling detection of novel disease genes in large cohorts inexpensively and rapidly (Choi et al., 2009; Ng et al., 2010). In CH, whole exome sequencing (WES) study in 27 unrelated CH families identified *WDR81* (OMIM# 617967) as a novel disease‐causing gene (Shaheen et al., 2017). Recently, our group performed a trio‐based WES in 177 CH probands—the majority being sporadic—and identified four novel CH risk genes (*TRIM71*, *SMARCC1*, *PTCH1*, and *SHH*) that account for ~10% of studied cases (Furey et al., 2018). Surprisingly, all four genes are known to regulate neural stem cell (NSC) proliferation and/or differentiation. However, these risk‐associated CH genes, when taken together, do not explain the etiology of most CH cases. We therefore hypothesized that new CH‐associated risk genes could be discovered by screening additional CH families using WES.

## 2 MATERIALS AND METHODS

### 2.1 Subjects

Two families containing one familial and one sporadic form of CH underwent WES. All study procedures and protocols comply with Yale University's Human Investigation Committee and Human Research Protection Program. Written informed consent for genetic studies was obtained from all participants.

### 2.2 Whole exome sequencing

Samples were sequenced at the Yale Center for Genome Analysis following the center's standard protocol. Targeted capture was performed using the Nimblegen SeqxCap EZ MedExome Target Enrichment Kit (Roche Sequencing), followed by 101 base paired‐end sequencing on the Illumina HiSeq 2000 instrument as previously described (Furey et al., 2018; Jin et al., 2017). Sequence metrics are shown in Supplementary Table S1. Sequence reads were mapped to the reference genome (GRCh37) with BWA‐MEM (Li & Durbin, 2010) and further processed using the GATK Best Practices workflows (McKenna et al., 2010; Van der Auwera et al., 2013), which include duplication marking, indel realignment and base quality recalibration. Single nucleotide variants and small indels were called with GATK HaplotypeCaller and annotated using ANNOVAR (Wang, Li, Li, & Hakonarson, 2010), dbSNP (v138), 1,000 Genomes (May 2013), NHLBI exome variant server, ExAC (v3), and gnomAD (v2.1.1) (Lek et al., 2016).

### 2.3 Kinship analysis

The relationship between the proband, sibling, and parents was estimated using the pairwise identify‐by‐descent calculation in PLINK (Purcell et al., 2007).

### 2.4 De novo variant calling and filtering

De novo variants were called using the TrioDeNovo program (Wei et al., 2015). De novo candidates were filtered based on the following hard filters: (a) have a minor allele frequency (MAF) ≤ 4 × 10^−4^ across all samples in ExAC, (b) pass GATK variant quality score recalibration, (c) have a minimum 10 reads total, five alternate allele reads and a 20% alternate allele ratio in proband, (d) have a minimum depth of 10 reference reads and alternate allele ratio <3% in parents, (e) are exonic or canonical splice‐site variants and (f) have a DQ ≥ 7 (suggested cutoff by authors of TrioDeNovo). Finally, false positives were excluded by in silico visualization using Integrative Genomics Viewer (Robinson et al., 2011) and BLAT search.

### 2.5 Dominant/recessive variant calling

Dominant variants were filtered for rareness (MAF ≤ 5 × 10^−5^ across all samples in 1,000 Genomes, exome variant server, and ExAC) and high‐quality heterozygotes (pass GATK VQSR, have a minimum of eight reads total, have a genotype quality score ≥20, and have alternate allele ratio ≥20%). The deleteriousness of missense mutations was predicted by the MetaSVM (Dong et al., 2015). We filtered recessive variants for rare (MAF ≤ 10^−3^ across all samples in 1,000 Genomes, exome variant server, and ExAC) homozygous and compound heterozygous mutations that exhibited high‐quality sequence reads (pass GATK VQSR, have a minimum of eight reads total for both proband and parents and have a genotype quality score ≥20). Only loss‐of‐function mutations (stop gain, stop loss, canonical splice‐site and, frameshift indels) and deleterious missense variants predicted by the MetaSVM algorithm (D‐Mis) were considered as potentially damaging.

### 2.6 Copy number variant analysis

To identify Copy number variants (CNVs) from WES data, the aligned reads were imported into XHMM (eXome‐Hidden Markov Model) (Fromer et al., 2012). Potential CNVs were inspected visually and prioritized based on genomic length, GC content of targets, and low sequence complexity. Finally, CNVs were validated by qPCR assays.

### 2.7 cDNA clones

The mouse KCC3a cDNA in the oocyte expression vector pBF was previously described (Ding, Ponce‐Coria, Ponce‐Coria, & Delpire, 2013). A c‐Myc epitope tag exists at the extreme NH_2_ terminus. To create the KCC3‐p.Pro605Leu mutant, we first subcloned a 466 bp *EcoRV*‐*EcoR*I fragment from full‐length mouse KCC3 cDNA into pBluescript. Using the QuikChange mutagenesis kit (Stratagene) and oligonucleotide primers 5′ ATCGCCAAGGATAACATCATACTCTTCCTTAGGGTTTTTGGTCAC 3′ and reverse, the codon encoding proline, CCT, was modified into CTC to encode for leucine. After sequence verification, the mutated fragment was reintroduced into the KCC3 cDNA to create the mutated cDNA clone.

### 2.8 Xenopus laevis

Oocytes were isolated as previously described (Delpire, Gagnon, Gagnon, Ledford, & Wallace, 2011) and in accordance with an approved Vanderbilt IACUC protocol. Small ovarian fragments were treated with collagenase D (4 × 90 min in 5 mg collagenase D/ml Ca^2+^‐free ND‐80 medium, Sigma) to isolate individual oocytes. Oocytes were then maintained overnight in modified L15 solution (250 ml Leibovitz L15 Ringer [Invitrogen], 200 ml deionized water, 952 mg HEPES [acid form], and 44 mg/L gentamycin [Invitrogen]; pH 7.4; 195 to 200 mOsM) at 16°C. The following day, groups of 25 oocytes were injected with 50 nl containing 15 ng KCC3 or mutant cRNA and returned to the incubator. Western blot analysis and ^86^Rb tracer flux studies to measure KCC3 cotransporter expression and function, respectively, were performed 3 days postinjection.

### 2.9 ^86^Rb uptake experiments

KCC3‐mediated K^+^ transport was measured through unidirectional ^86^Rb uptakes in groups of 20–25 oocytes placed in 35‐mm dishes. Oocytes were washed once with 3 ml isosmotic saline (96 mM N‐methylglucamine‐Cl, 4 mM KCl, 2 mM CaCl_2_, 1 mM MgCl_2_, 5 mM HEPES, pH 7.4, 200 mOsM) and preincubated for 15 min in 1 ml identical solution or hypotonic solution (140 mOsM) containing 1 mM ouabain. The solution was then aspirated and replaced with 1 ml isosmotic or hyposmotic flux solution containing 5 μCi ^86^Rb. Two 5 μl samples of flux solution were taken at the beginning of each uptake period, placed in scintillation vials, and used as standards. After 1 hr uptake, the radioactive solution was aspirated and the oocytes were washed four times with 3 ml ice‐cold isosmotic or hyperosmotic solution. Single oocytes were then transferred into glass vials, lysed for 1 hr with 200 μl 0.25 N NaOH, and neutralized with 100 μl glacial acetic acid. ^86^Rb activity was measured by β‐scintillation counting. K^+^ influx was expressed in picomoles K^+^ per oocyte per hour.

For Western blot analysis, groups of eight oocytes were homogenized with pipet in 200 μl lysis buffer containing 150 mM NaCl, 50 mM TRIS pH 8.5, 2 mM EDTA, 0.1% SDS, 0.5% Na‐deoxycholate, 1% CHAPS, and incubated on ice for 30 min. The samples were then spun at maximum speed at 4°C for 20 min and the supernatant was collected. Samples (25 μl) were added to 25 μl 4× sample buffer + DTT and denatured at 70°C for 15 min. After separation on 7.5% SDS‐PAGE gel, the gel was transferred to polyvinylidene difluoride (PVDF) membrane that was incubated overnight with anti‐c‐Myc antibody (mouse monoclonal, clone 9E10 from Thermo Fisher).

## 3 RESULTS

**Figure 1:**
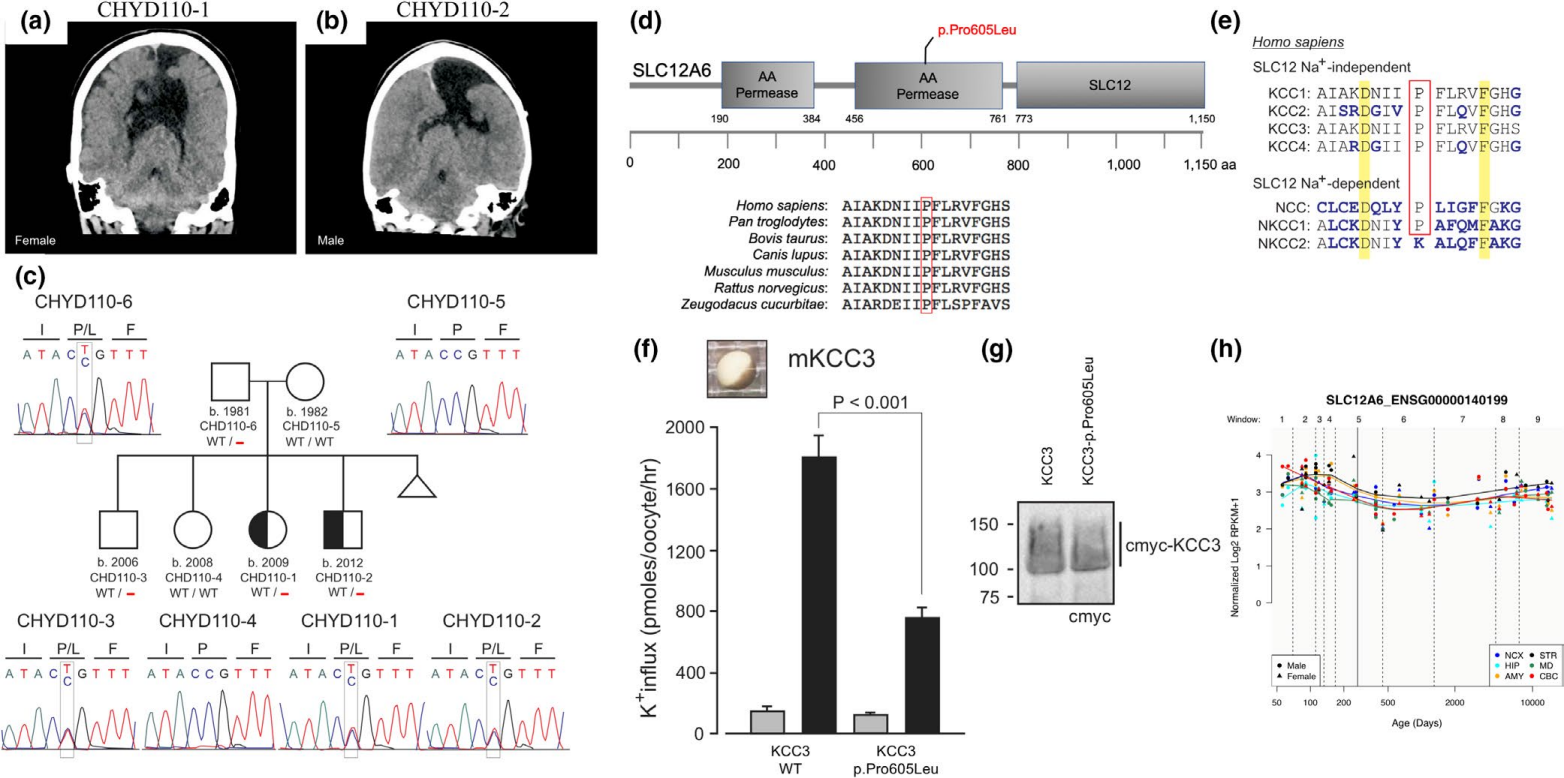
Identification of a novel inherited *SLC12A6* (*KCC3*) mutation in familial CH with aqueductal stenosis. Coronal head computed tomography images in (a) proband CHYD110‐1 and (b) affected sibling CHYD110‐2. Note ventriculomegaly, agenesis of the corpus callosum, and schizencephaly. (c) Pedigree and DNA chromatograms depicting a heterozygous c.C1814T (p.Pro605Leu) mutation in *SLC12A6*, encoding the K^+^‐Cl^−^ cotransporter KCC3, in the indicated individuals. Triangle represents a spontaneous abortion that occurred after the birth of the two affected children. (d) Evolutionary conservation of *KCC3* p.Pro605 across multiple species and (e) among human *N*(K)CC family transporters (KCC1‐4). (f) Decreased function of KCC3 p.Pro605Leu transporter. Left, K^+^ influx in oocytes injected with WT or p.Pro605Leu mouse KCC3 cRNA. Flux was measured in isotonic (gray) or hypotonic (black) activating conditions. Bars represent mean ± *SEM*, *n* = 24 oocytes. Difference between black bars is highly significant (*p* < .001, ANOVA). (g) Western blot showing expression of c‐myc‐tagged WT and mutant KCC3 in oocyte membrane fractions. Primary antibody was directed against c‐myc epitope. (h) Spatial‐temporal gene expression for *SLC12A6* in the brain development process across several brain regions using the bulk RNA‐sequencing data from the PsychENCODE project. The *x*‐axis denotes postconception days. The *y*‐axis denotes normalized expression level (represented by log2 of RPKM). NCX: Neocortex; HIP: Hippocampus; AMY: Amygdala; STR: Striatum; MD: Mediodorsal nucleus of the thalamus; CBC: Cerebellar cortex

In kindred CHYD110, both the female proband (CHYD110‐1) and the affected male sibling (CHYD110‐2), born to unaffected parents, were prenatally diagnosed with ventriculomegaly, treated with ventriculoperitoneal CSF shunting, and exhibited neurodevelopmental delay. Each had similar marked obstructive hydrocephalus and aqueductal stenosis, agenesis of the corpus callosum, and schizencephaly (Figure 1a,b). Neither child had signs of peripheral sensorimotor neuropathy (Kahle, Flores, et al., 2016). Furthermore, the mother had an abortion due to fetus diagnosed with fetal hydrocephalus. Results from comparative genomic hybridization microarrays were negative, and no CNVs were detected that segregated with both affected probands. In contrast, a single inherited heterozygous D‐Mis mutation (c.C1814T [p.Pro605Leu]) in *SLC12A6*, encoding the KCC3 K^+^‐Cl^−^ transporter, was identified in both affected probands and met the criteria for being a predicted damaging mutation in a mutation‐intolerant gene (mis‐*Z* = 2.96 in gnomAD) (Lek et al., 2016; Figure 1c). *KCC3* p.Pro605Leu mutation showed incomplete penetrance, as an unaffected sibling (CHYD110‐3) also carried this mutation. p.Pro605 is conserved among KCC3 orthologs and human KCC paralogs, and maps to the critical KCC3 permease domain (Figure 1d,e). p.Pro605Leu is extremely rare in all public databases (ExAC MAF = 0; gnomAD MAF = 4.0 × 10^−6^), and deleterious by the MetaSVM (Dong et al., 2015) and CADD (score = 34 [v1.3]) (Kircher et al., 2014) algorithms. *Xenopus* oocyte experiments suggest that this mutation reduces KCC3‐dependent K^+^ transport compared to wild type KCC3 (*p* < .001, *n* = 24; Figure 1f), but has no effect on transporter surface expression (Figure 1g). Interregional transcriptome analysis using bulk RNA‐sequencing data from the PsychENCODE project (Li et al., 2018) showed that KCC3 is highly expressed in early brain development across multiple regions (Figure 1h).

**Figure 2:**
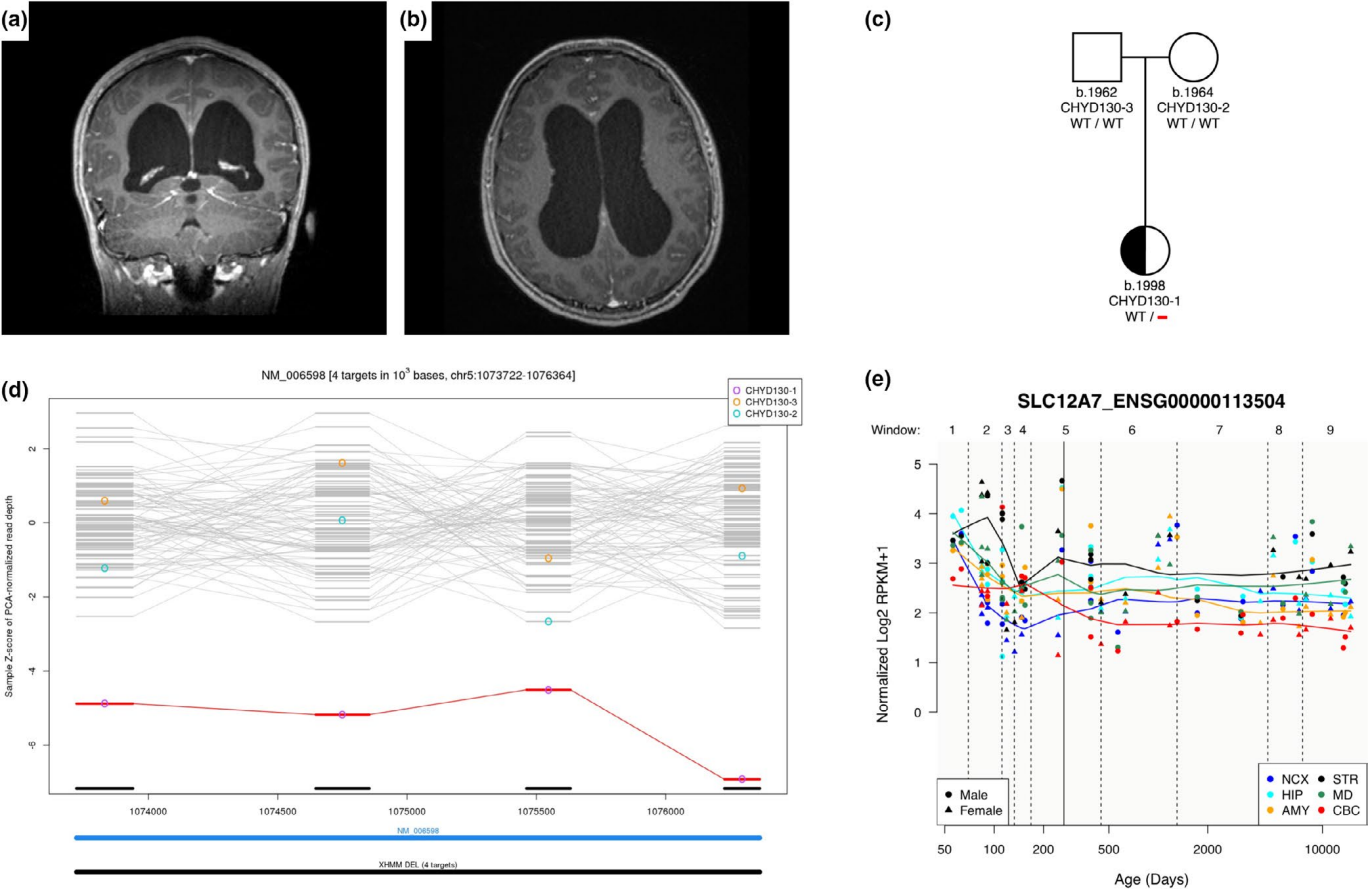
Identification of a novel de novo* SLC12A7* (*KCC4*) deletion in sporadic CH with aqueductal stenosis. Coronal (a) and axial (b) magnetic resonance images in proband CHYD130‐1 demonstrating marked ventriculomegaly. (c) Pedigree depicting a heterozygous de novo deletion in *SLC12A7*, encoding the KCC3 paralog KCC4, which is present in CHYD130‐1 but absent in his unaffected parents. (d) XHMM plot of exome sequencing data demonstrating de novo copy number deletion which expands four targets from chr5:1073722 to chr5:1076364. (e) Spatial‐temporal gene expression for *SLC12A7* in the brain development process across several brain regions using the bulk RNA‐sequencing data from the PsychENCODE project. The *x*‐axis denotes postconception days. The *y*‐axis denotes normalized expression level (represented by log2 of RPKM). NCX: Neocortex; HIP: Hippocampus; AMY: Amygdala; STR: Striatum; MD: Mediodorsal nucleus of the thalamus; CBC: Cerebellar cortex

In kindred CHYD130, male proband CHYD130‐1 of unaffected parents was diagnosed with obstructive hydrocephalus and aqueductal stenosis in the setting of progressive headaches, and was treated with endoscopic third ventriculostomy (Figure 2a,b). The child had no signs or symptoms of kidney or inner ear dysfunction (Boettger et al., 2002). WES revealed no protein‐damaging de novo or rare inherited pathologic single nucleotide variants. In contrast, a novel de novo CNV (deletion) was detected in *SLC12A7*, encoding the KCC3 paralog KCC4. qPCR assays validated the presence of this deletion in the proband but not in the parents. Strikingly, no *SLC12A7* CNVs were detected in 60,706 subjects in the ExAC Browser as well as 1,789 control trios comprising parents and unaffected siblings of autism probands from the Simons Simplex Collection previously analyzed. (Jin et al., 2017; Lek et al., 2016). These data suggest that insertions or duplications in this gene are under strong purifying selection in the general population. Further, when we searched results for “5:1073722–1076364” in the DECIPHER database which contains genomic and clinical data from 29,742 patients with rare diseases, there were 68 unrelated patients with a CNV covering this region (Supplementary Table S2). Among these 68 patients, 54 (79.4%) were CNV losses, of whom 27 (50%) had a description of clinical phenotypes. Interestingly, 21 of these 27 patients had neurodevelopment disorders, cognitive impairment, or intellectual disability, including six with microcephaly, suggesting genes within these CNVs might contribute to brain growth or development. Although most of these CNVs were longer than our discovered de novo CNV deletion (2.6kb) and contain multiple genes, we could not rule out the possibility that *SLC12A7* is the culprit for these patients. Interregional transcriptome analysis from the PsychENCODE project (Li et al., 2018) showed KCC4 is expressed in early brain development across multiple regions (Figure 2d).

## 4 DISCUSSION

Our genomic analyses and functional experiments suggest that mutations in *SLC12A6* and *SLC12A7* contribute to the CH phenotype. To our knowledge, these are the first ion transporter mutations associated with human CH. However, the small number of observations, and lack of replication studies and robust animal experiments are limitations of this study. Further studies are required to establish causality.

Protein‐truncating homozygous *KCC3* mutations cause Autosomal Recessive Agenesis of the Corpus Callosum with Peripheral Neuropathy (Howard et al., 2002) (ACCPN; OMIM# 218000). ACCPN patients have been reported with increased head circumference (i.e., macrocephaly) and stenosis of the aqueduct of Sylvius (Howard et al., 2002). In contrast, de novo gain‐of‐function point mutations in *KCC3* cause early onset, progressive, and severe peripheral neuropathy primarily affecting motor neurons (Kahle, Flores, et al., 2016). We noted that our patients with *KCC3* mutation had no apparent signs of peripheral neuropathy, but did have agenesis of the corpus callosum. No pathogenic *KCC4* mutations have been reported in humans to date*.* Our data support the known phenotypic heterogeneity, incomplete penetrance, and variable expressivity of *SLC12A* mutations (Howard et al., 2002), features which are also seen with mutations in *L1CAM* and other recently discovered *bona fide* CH genes (Furey et al., 2018).

*SLC12A* family paralogs KCC3 and KCC4 form hetero‐oligomeric assemblies (Simard et al., 2007); however, the actual structure of the complex and the interaction mechanism remains unknown. Through structural studies of other members in KCC family, it is possible that KCC3 and KCC4 hetero‐oligomer assembly involves C‐terminal domain of KCC4, disulfide bridges or other unknown mechanisms (Agez et al., 2017). We speculate that heterozygous *SLC12A6* p.Pro605Leu and *SLC12A7* deletion could have hypomorphic effects and/or impair the function of multimeric KCC3/4 complexes.

KCC3 and KCC4 localize to the CSF‐producing choroid plexus and NSCs in the developing brain (Simard et al., 2007). The observed CH phenotypes in the patients described might result from impaired Cl^−^‐dependent K^+^ homeostasis that impacts NSC fate (Cui et al., 2017; Liebau et al., 2007) and/or CSF secretion from the choroid plexus. Together, these compelling results should spur further functional investigation into the role of KCC3/4 in brain development and CH pathogenesis in vivo.

## CONFLICT OF INTEREST

The authors have no potential conflicts of interest to disclose.
