# Supplementary material for: SLC12A ion transporter mutations in sporadic and familial human congenital hydrocephalus
Source: Mol Genet Genomic Med. 2019 Aug 8;7(9):e892. doi: 10.1002/mgg3.892 (PMC6732308; doi:10.1002/mgg3.892)
Supplement: Supplementary file 1 [file MGG3-7-e892-s001.docx]

| **Supplementary Table 1**. Summary sequencing statistics for two hydrocephalus families | |
| --- | --- |
| Category | Cases  (MedExome; N=7) |
| Read length (bp) | 101 |
| # of reads per sample (M) | 46.79 |
| Median coverage at each targeted base (X) | 34 |
| Mean coverage at each targeted base (X) | 40.23 |
| % of all reads that map to target | 49.46% |
| % of all bases that map to target | 40.04% |
| % of targeted bases read at least 8x | 96.03% |
| % of targeted bases read at least 10x | 94.49% |
| % of targeted bases read at least 15x | 88.59% |
| % Mean error rate | 0.28% |

| **Supplementary Table 2. Clinical and genomic information for 68 DECIPHER patients in search for “5:1073722-1076364”** | | | | | | |
| --- | --- | --- | --- | --- | --- | --- |
| **DECIPHER ID** | **Variant** | **Sex** | **Size** | **Pathogenicity/Contribution** | **Inheritance** | **Phenotype(s)** |
| 4119 | 5:204849-6753953 | other | 6.55 Mb | [Unknown](https://decipher.sanger.ac.uk/info/pathogenicity) | Unknown | Adrenogenital syndrome, Aganglionic megacolon, Aplasia/Hypoplasia of the breasts, Frontal balding, Generalized hirsutism, Hypotelorism, Intellectual disability, Intention tremor, Kyphosis, Mitral regurgitation, Patent ductus arteriosus, Prematurely aged appearance, Proportionate short stature, Scoliosis, Seizures, Short palm, Synophrys, Uterine neoplasm |
|  | Triplication |  |  |  |  |  |
| 252489 | 5:1062209-1364929 | 46XY | 302.72 kb | Unknown | Unknown | Atrioventricular canal defect, Cleft palate, Cryptorchidism, Micropenis |
|  | Duplication |  |  |  |  |  |
| 252490 | 5:71704-4287158 | 46XY | 4.22 Mb | Unknown | Unknown | Autism, Cognitive impairment |
|  | Deletion |  |  |  |  |  |
| 252526 | 5:95243-8142095 | 46XX | 8.05 Mb | Unknown | De novo constitutive |  |
|  | Deletion |  |  |  |  |  |
| 252530 | 5:95243-7144625 | 46XY | 7.05 Mb | Unknown | De novo constitutive |  |
|  | Deletion |  |  |  |  |  |
| 253159 | 5:95243-18909607 | 46XY | 18.81 Mb | Unknown | De novo constitutive |  |
|  | Deletion |  |  |  |  |  |
| 253160 | 5:95243-8726021 | 46XY | 8.63 Mb | Unknown | De novo constitutive |  |
|  | Deletion |  |  |  |  |  |
| 254052 | 5:95243-16154654 | 46XX | 16.06 Mb | Unknown | Unknown |  |
|  | Deletion |  |  |  |  |  |
| 256304 | 5:130931-36780974 | other | 36.65 Mb | Unknown | Imbalance arising from a balanced parental rearrangement | Atrioventricular canal defect |
|  | Deletion |  |  |  |  |  |
| 256382 | 5:151537-24098448 | 46XX | 23.95 Mb | Unknown | Unknown |  |
|  | Deletion |  |  |  |  |  |
| 257265 | 5:95243-4147304 | 46XX | 4.05 Mb | Unknown | De novo constitutive |  |
|  | Deletion |  |  |  |  |  |
| 257513 | 5:95243-24001674 | 46XY | 23.91 Mb | Unknown | Unknown |  |
|  | Deletion |  |  |  |  |  |
| 258252 | 5:95302-11199684 | 46XX | 11.10 Mb | Unknown | Unknown | Cognitive impairment, High pitched voice |
|  | Deletion |  |  |  |  |  |
| 259299 | 5:95243-21674533 | 46XY | 21.58 Mb | Unknown | Imbalance arising from a balanced parental rearrangement | Hearing impairment, High palate, Hydronephrosis, Hypoplasia of the corpus callosum, Muscular hypotonia, Optic disc hypoplasia, Ureteral atresia, Ventricular septal defect |
|  | Deletion |  |  |  |  |  |
| 262907 | 5:22179-15851456 | 46XY | 15.83 Mb | Unknown | Unknown | Clinodactyly of the 5th finger, Deeply set eye, Hypertelorism, Intellectual disability, Myopia, Prominent supraorbital ridges, Psychosis, Short nose, Vesicoureteral reflux |
|  | Duplication |  |  |  |  |  |
| 263168 | 5:151737-29760922 | 46XX | 29.61 Mb | Unknown | Unknown |  |
|  | Deletion |  |  |  |  |  |
| 264192 | 5:88308-8328842 | 46XX | 8.24 Mb | Unknown | Unknown | Generalized seizures, Global developmental delay, Moderately short stature, Poor speech |
|  | Duplication |  |  |  |  |  |
| 268387 | 5:25942-5307253 | 46XY | 5.28 Mb | Unknown | Imbalance arising from a balanced parental rearrangement | Broad forehead, Intellectual disability, Single transverse palmar crease |
|  | Deletion |  |  |  |  |  |
| 268567 | 5:25942-30067642 | 46XX | 30.04 Mb | Unknown | Unknown |  |
|  | Deletion |  |  |  |  |  |
| 268568 | 5:25942-25196251 | 46XY | 25.17 Mb | Unknown | Unknown | Autism, Intellectual disability, Ptosis, Sleep disturbance |
|  | Duplication |  |  |  |  |  |
| 269509 | 5:71704-35463036 | 46XX | 35.39 Mb | Unknown | De novo constitutive | Autism, Bruxism, Delayed speech and language development, Focal seizures with impairment of consciousness or awareness, Generalized tonic seizures, Intellectual disability, Stereotypy |
|  | Duplication |  |  |  |  |  |
| 270177 | 5:22179-8659713 | 46XX | 8.64 Mb | Unknown | De novo constitutive | Intellectual disability |
|  | Deletion |  |  |  |  |  |
| 270697 | 5:151677-4580664 | 46XY | 4.43 Mb | Unknown | De novo constitutive | Arachnoid cyst, Cerebellar vermis hypoplasia, Duodenal atresia, Intellectual disability, Microcephaly |
|  | Deletion |  |  |  |  |  |
| 271094 | 5:151737-31162900 | 46XX | 31.01 Mb | Unknown | Unknown |  |
|  | Duplication |  |  |  |  |  |
| 272148 | 5:1-21486661 | 46XX | 21.49 Mb | Unknown | Imbalance arising from a balanced parental rearrangement | Abnormality of the foot, Abnormality of the inner ear, Atrial septal defect, Pleural effusion, Ventricular septal defect |
|  | Duplication |  |  |  |  |  |
| 272301 | 5:122963-8145689 | 46XX | 8.02 Mb | Unknown | De novo constitutive | Complex febrile seizures, Feeding difficulties in infancy, Gastroesophageal reflux, Intellectual disability, moderate, Periventricular gray matter heterotopia, Ventricular septal defect |
|  | Deletion |  |  |  |  |  |
| 272343 | 5:21949-5471486 | 46XX | 5.45 Mb | Unknown | Inherited from parent with similar phenotype to child |  |
|  | Deletion |  |  |  |  |  |
| 272684 | 5:151737-22246071 | 46XX | 22.09 Mb | Unknown | Unknown | Abnormal heart morphology, Feeding difficulties in infancy |
|  | Deletion |  |  |  |  |  |
| 274949 | 5:801915-1705456 | 46XX | 903.54 kb | Unknown | De novo constitutive |  |
|  | Duplication |  |  |  |  |  |
| 275837 | 5:113576-7580683 | 46XX | 7.47 Mb | Unknown | Unknown |  |
|  | Deletion |  |  |  |  |  |
| 276535 | 5:95243-10972789 | 46XY | 10.88 Mb | Unknown | Unknown | Attention deficit hyperactivity disorder, Brachycephaly, Intellectual disability, severe, Microcephaly, Self-injurious behavior |
|  | Deletion |  |  |  |  |  |
| 279259 | 5:26142-12800210 | 46XY | 12.77 Mb | Unknown | De novo constitutive | Ataxia, Bilateral ptosis, Cryptorchidism, Delayed speech and language development, Microcephaly |
|  | Deletion |  |  |  |  |  |
| 279535 | 5:25942-21269598 | 46XY | 21.24 Mb | Unknown | Unknown |  |
|  | Deletion |  |  |  |  |  |
| 279916 | 5:35779-13750112 | 46XX | 13.71 Mb | Unknown | De novo constitutive | Abnormality of the face, Global developmental delay |
|  | Deletion |  |  |  |  |  |
| 280719 | 5:95243-2475339 | 46XY | 2.38 Mb | Unknown | De novo constitutive |  |
|  | Deletion |  |  |  |  |  |
| 280891 | 5:95243-5608155 | 46XX | 5.51 Mb | Unknown | De novo constitutive | Abnormal facial shape, Global developmental delay |
|  | Deletion |  |  |  |  |  |
| 282379 | 5:151737-5343225 | 46XY | 5.19 Mb | Unknown | Unknown | Absent speech, Hypospadias, Intrauterine growth retardation, Pierre-Robin sequence |
|  | Deletion |  |  |  |  |  |
| 283147 | 5:151737-16207386 | 46XX | 16.06 Mb | Unknown | Unknown | Intellectual disability, moderate, Microcephaly |
|  | Deletion |  |  |  |  |  |
| 283397 | 5:1-22246041 | 46XX | 22.25 Mb | Pathogenic | Unknown |  |
|  | Deletion |  |  |  |  |  |
| 284051 | 5:10001-22825583 | 46XY | 22.82 Mb | Pathogenic | De novo constitutive | Abnormal facial shape, Atrial septal defect, Bilateral cryptorchidism, Clinodactyly of the 5th finger, Congenital microcephaly, Intrauterine growth retardation, Long fingers, Long toe, Patent ductus arteriosus |
|  | Deletion |  |  |  |  |  |
| 285458 | 5:38129-6257996 | 46XX | 6.22 Mb | Pathogenic | Unknown |  |
|  | Deletion |  |  |  |  |  |
| 285961 | 5:204849-3876457 | 46XY | 3.67 Mb | Pathogenic | Paternally inherited, constitutive in father | Anteverted nares, Congenital conductive hearing impairment, Depressed nasal bridge, Global developmental delay, Motor delay, Short nose, Thick upper lip vermilion |
|  | Deletion |  |  |  |  |  |
| 286019 | 5:257649-14064732 | 46XX | 13.81 Mb | Pathogenic | De novo constitutive | Autism, Broad forehead, Diastema, Generalized joint laxity, Global developmental delay, Micrognathia, Self-injurious behavior, Wide nose |
|  | Deletion |  |  |  |  |  |
| 287455 | 5:22149-8026308 | 46XX | 8.00 Mb | Likely pathogenic | De novo constitutive |  |
|  | Deletion |  |  |  |  |  |
| 288568 | 5:919451-1248468 | unknown | 329.02 kb | Likely benign | Paternally inherited, constitutive in father | Blepharophimosis |
|  | Duplication |  |  |  |  |  |
| 289314 | 5:16497-19869036 | unknown | 19.85 Mb | Uncertain | Unknown | Feeding difficulties in infancy, Generalized hypotonia, Hypertelorism |
|  | Deletion |  |  |  |  |  |
| 292369 | 5:21949-1259044 | 46XX | 1.24 Mb | Uncertain | De novo constitutive |  |
|  | Deletion |  |  | Partial |  |  |
| 294745 | 5:113576-4970954 | 46XX | 4.86 Mb | Unknown | Unknown | Decreased body weight, Epicanthus, High pitched voice, Hypertelorism, Large forehead, Pointed chin, Short stature, Strabismus, Wide nasal bridge |
|  | Deletion |  |  |  |  |  |
| 294787 | 5:74149-4633601 | 46XY | 4.56 Mb | Likely pathogenic | Unknown |  |
|  | Deletion |  |  |  |  |  |
| 295116 | 5:113576-4612673 | 46XX | 4.50 Mb | Unknown | Unknown | Abnormal facial shape, Intellectual disability |
|  | Deletion |  |  |  |  |  |
| 295221 | 5:1-10124257 | 46XY | 10.12 Mb | Pathogenic | Unknown |  |
|  | Deletion |  |  | Full |  |  |
| 305564 | 5:22178-8337920 | 46XY | 8.32 Mb | Unknown | Unknown |  |
|  | Deletion |  |  |  |  |  |
| 306296 | 5:151737-18215676 | unknown | 18.06 Mb | Uncertain | Unknown |  |
|  | Deletion |  |  |  |  |  |
| 306822 | 5:151737-17425722 | unknown | 17.27 Mb | Uncertain | Unknown |  |
|  | Deletion |  |  |  |  |  |
| 314141 | 5:686315-1527676 | 46XX | 841.36 kb | Uncertain | Unknown | Abnormality of the periorbital region, Papillary thyroid carcinoma, Periorbital edema, Renal angiomyolipoma, Renal cell carcinoma |
|  | Duplication |  |  | Uncertain |  |  |
| 314621 | 5:1-42573850 | 46XX | 42.57 Mb | Pathogenic | Imbalance arising from a balanced parental rearrangement |  |
|  | Duplication |  |  | Full |  |  |
| 314863 | 5:151737-5956060 | 46XY | 5.80 Mb | Unknown | Paternally inherited, constitutive in father |  |
|  | Deletion |  |  |  |  |  |
| 322792 | 5:151737-10390486 | 46XX | 10.24 Mb | Likely pathogenic | Unknown | Abnormal fear/anxiety-related behavior, Intellectual disability, moderate |
|  | Deletion |  |  | Uncertain |  |  |
| 326556 | 5:26142-2453629 | 46XY | 2.43 Mb | Pathogenic | Imbalance arising from a balanced parental rearrangement |  |
|  | Deletion |  |  | Partial |  |  |
| 326667 | 5:151737-18997054 | 46XY | 18.85 Mb | Likely pathogenic | Unknown | Cat cry |
|  | Deletion |  |  | Full |  |  |
| 327403 | 5:151737-1340609 | 46XX | 1.19 Mb | Pathogenic | De novo constitutive |  |
|  | Deletion |  |  | Partial |  |  |
| 328436 | 5:113576-12747875 | 46XX | 12.63 Mb | Pathogenic | Unknown | Abnormal facial shape, Abnormal heart morphology, Asymmetric short stature, Global developmental delay |
|  | Deletion |  |  | Full |  |  |
| 331049 | 5:697770-1478459 | 46XY | 780.69 kb | Likely pathogenic | Paternally inherited, constitutive in father | Autistic behavior |
|  | Duplication |  |  |  |  |  |
| 339222 | 5:182568-5088435 | 46XX | 4.91 Mb | Uncertain | De novo constitutive | Cerebral hemorrhage, Hearing impairment, Neurodevelopmental delay, Strabismus |
|  | Triplication |  |  | Uncertain |  |  |
| 340213 | 5:113576-3792922 | 46XX | 3.68 Mb | Likely pathogenic | Unknown |  |
|  | Deletion |  |  | Full |  |  |
| 346703 | 5:151737-11460236 | unknown | 11.31 Mb | Pathogenic | De novo constitutive | Global developmental delay, High pitched voice, Strabismus |
|  | Deletion |  |  |  |  |  |
| 369296 | 5:530786-1564878 | 46XY | 1.03 Mb | Likely pathogenic | Maternally inherited, constitutive in mother | Microcephaly |
|  | Deletion |  |  | Full |  |  |
| 379305 | 5:151737-10464853 | 46XY | 10.31 Mb | Pathogenic | Unknown | Autism, Global developmental delay |
|  | Deletion |  |  |  |  |  |

DECIPHER Browser: <https://decipher.sanger.ac.uk>
